# Preliminary clinical evaluation of guided bone regeneration using carbonate apatite granules and poly(lactic acid/caprolactone) membranes: a prospective interventional study

**DOI:** 10.1186/s40729-026-00687-1

**Published:** 2026-05-08

**Authors:** Akira Takahashi, Yoichiro Ogino, Tatsuya Matsuzaki, Masafumi Kihara, Satomi Wachi, Tomotaka Sugi, Yuma Hashiguchi, Kiyoshi Koyano, Yasunori Ayukawa

**Affiliations:** 1 Kyushu University, Fukuoka, Japan; 2 Kyushu University Hospital, Fukuoka, Japan

**Keywords:** Guided bone regeneration, Carbonate apatite, Poly(lactic acid/caprolactone) membrane

## Abstract

**Purpose:**

This prospective clinical study aimed to evaluate the effectiveness and clinical applicability of guided bone regeneration (GBR) using a combination of carbonate apatite granules and poly(lactic acid/caprolactone) (PLCL) membranes in patients with alveolar bone deficiencies.

**Methods:**

A single-arm interventional study was conducted involving 16 participants and 18 grafted sites. GBR was performed using carbonate apatite granules mixed with saline and covered with PLCL membranes. Radiopaque markers were placed at the grafted sites to enable standardized radiographic evaluation. Pre- and post-operative computed tomography (CT) scans were analyzed to assess changes in crestal bone width and cross-sectional bone area. Postoperative complications were monitored at multiple time points, and statistical analyses were performed to evaluate bone augmentation outcomes.

**Results:**

Radiographic analysis revealed a statistically significant increase in crestal bone width and cross-sectional bone area following GBR (*P* < 0.01). Minor wound dehiscence was observed in 33.3% of cases at 2 weeks and 22.2% at 1 month, but no infections occurred at any time point. At 3 and 6 months, all sites showed uneventful healing. The combination of carbonate apatite and PLCL membranes demonstrated favorable biocompatibility and mechanical stability, contributing to predictable bone augmentation.

**Conclusions:**

GBR using carbonate apatite granules and PLCL membranes resulted in significant bone regeneration with low complication rates. This synthetic material combination may offer a reliable approach for GBR. Further studies with larger cohorts and longer follow-up are recommended to validate long-term outcomes and clinical relevance.

## Background

Over the past decades, guided bone regeneration (GBR) has been widely recognized as a standard therapeutic approach for ridge preservation and bone augmentation in the management of peri-implant bone deficiencies [1–3]. Barrier membranes are essentially applied as a physical barrier to maintain space for bone regeneration in GBR [1–4]. These membranes also have an important role to prevent soft tissue ingrowth [4]. GBR has been introduced and widely applied in the field of implant dentistry to obtain sufficient bone volume essential for the long-term success of implant treatment [3–5]. An adequate clinical environment for implant placement, either following or combined with GBR, can contribute to predictable outcomes in implant treatment [6–9]. However, it is widely recognized that various factors can influence the outcomes of GBR and subsequent implant treatment [8, 10–12]. One of these factors is materials used for GBR.

Modern GBR techniques employ a range of membrane types (non-resorbable and resorbable) with or without bone graft materials. Regarding non-resorbable barrier membranes, expanded polytetrafluoroethylene (ePTFE) membranes reinforced with titanium have been widely used due to their mechanical stability, superior space-maintaining capacity, biocompatibility, and proven efficacy in promoting bone regeneration [2, 13]. Despite these advantages, non-resorbable membranes are associated with two significant limitations in clinical practice. Their stiffness can induce soft tissue dehiscence, resulting in membrane exposure [14]. Additionally, a second surgical intervention is required for membrane removal [1–4]. Compared to non-resorbable membranes, resorbable alternatives are commonly selected for their hydrophilic nature, offering improved manageability and ease of use during surgery [1–4]. In addition to barrier membranes, the application of bone graft materials is closely related to the regenerative outcome of GBR. Autografts, allografts, and xenografts have been widely utilized as bone graft materials, and their effects on bone regeneration and implant treatment outcomes have been evaluated [4, 8, 15]. Based on these backgrounds, the use of synthetic materials in GBR also has been extensively investigated [16]. In Japan, carbonate apatite granules have been used for bone augmentation in implant dentistry [17–19], as they were clinically approved based on these trial data and have since become a widely accepted material in this field. Regarding synthetic barrier membranes, a synthetic copolymer of poly(lactic acid/caprolactone) (PLCL) was developed as a bilayer membrane with favorable biological and mechanical properties, as demonstrated in preclinical studies [20, 21]. As the demand for predictable and minimally invasive GBR techniques grows, synthetic materials that combine favorable biological and mechanical properties are increasingly being explored. The combination of carbonate apatite granules and PLCL membranes may fulfill these requirements.

This pilot study aimed to evaluate the clinical effectiveness of GBR using a combination of carbonate apatite granules and PLCL membranes, and to explore their potential clinical applicability.

## Methods

### Ethical approval

This study was reviewed and approved by the Kyushu University Hospital Clinical Research Review Board (CRB7180005) and subsequently registered with the Japan Registry of Clinical Trials (jRCTs072190038) prior to commencement. The study was conducted in accordance with the guidelines of the Declaration of Helsinki.

### Study design and participants

This study was designed as a single-arm prospective interventional study to evaluate the feasibility and early clinical performance of GBR using CO₃Ap granules and a PLCL membrane. As a preliminary study, the sample size was determined based on feasibility rather than a formal statistical calculation. In addition, the COVID‑19 pandemic caused substantial delays in patient recruitment and follow‑up, resulting in an extended study period from the original plan.

The candidates of study participants were patients with bone concavities or reduced bone width who had been adequately informed about the study and had voluntarily provided written consent.

**Fig. 1:**
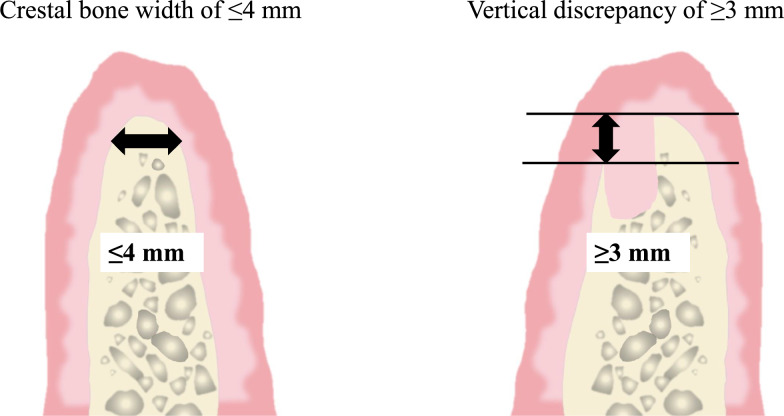
Schematic representation of alveolar bone morphology meeting the inclusion criteria. The schemas represent alveolar ridges with a crestal bone width of ≤ 4 mm and a vertical discrepancy of ≥ 3 mm, which are defined as inclusion criteria for guided bone regeneration in this study

The detailed inclusion criteria were as follows:

1. Patients aged 20 years or older.
2. Patients eligible for oral surgical procedures.
3. Patients exhibiting bony concavities on the buccal or lingual (palatal) side as confirmed by preoperative imaging (alveolar ridges with a crestal bone width of ≤ 4 mm, or a vertical discrepancy of ≥ 3 mm between the buccal and lingual cortical bone heights) (Fig. 1).
4. Patients without evident infection or gingival dehiscence at the site designated for GBR.

The detailed exclusion criteria were as follows:

1. Patients ineligible for oral surgical procedures due to systemic conditions.
2. Patients who are pregnant or may be pregnant.
3. Patients undergoing simultaneous implant placement and GBR.
4. Patients who are considered inappropriate for study participation by investigators.

A sample size of 20 patients was selected based on practical considerations and the availability of eligible cases during the study period. There is no standardized interval between tooth extraction and GBR. In this study, the timing of GBR was determined based on the clinical condition of each site and the judgment of the treating surgeon.

### Study schedule

The study schedule is illustrated in Table 1. Based on the study schedule, the subsequent description summarizes the key components of the research.

**Table 1:** Study schedule with timing constraints

Visit	Description	Timing constraint
Visit 0	Informed consent	
Visit 1	Preoperative examination (blood tests and CT scan)	1 month ± 14 days before GBR surgery
Visit 2	Enrollment and surgical explanation	Between Visit 1 and Visit 3
Visit 3	GBR operation	
Visit 4–6	Intraoral observation	Visit 4: 10 ± 4 days after Visit 3 Visit 5: 1 month ± 7 days after Visit 3 Visit 6: 3 months ± 7 days after Visit 3
Visit 7	Final assessment (CT scan)	6 ± 1 months after Visit 3

CT: computed tomography, GBR: guided bone regeneration

### Informed consent

Prior to enrollment in this study, all candidates were provided with written informed consent documents and signed the consent form.

### Preoperative examination

**Fig. 2:**
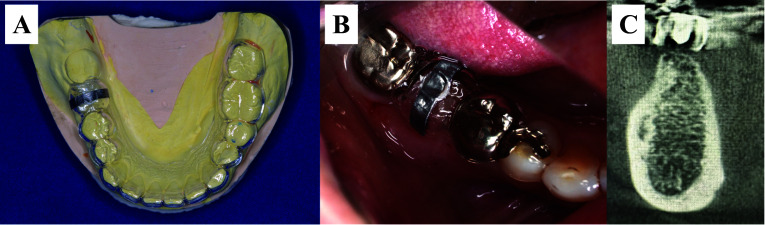
An example of radiopaque marker. **A** Radiopaque marker attached to the dental cast, with a circumferential lead foil around the crown and a vertical marker positioned perpendicular to the occlusal plane. **B** Intraoral placement of the radiopaque marker. **C** CT image showing the radiopaque marker clearly visible around the crown, with the vertical component facilitating three‑dimensional image alignment

Prior to the GBR procedure, blood tests were performed to assess the patients’ systemic health status, and a computed tomography (CT) scan with a radiopaque marker (Fig. 2) was performed to assess bone morphology and determine eligibility criteria. The same marker, placed at GBR site, was used for both pre- and post-operative scans, enabling precise three-dimensional evaluation of bone changes over time.

### GBR operation and surgical procedure

**Fig. 3:**
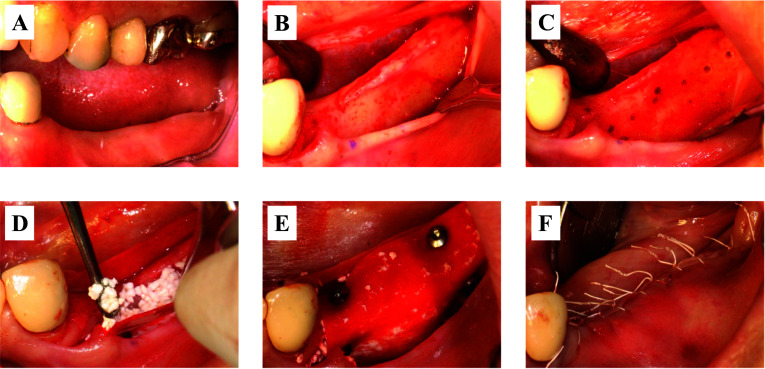
Surgical procedure of GBR using carbonate apatite granules and a PLCL membrane. **A** Preoperative intraoral image. **B** Mucoperiosteal flap elevation following crestal incision, exposing the alveolar bone surface. **C** Decortication to induce bleeding. **D** Graft of carbonate apatite granules at GBR site. **E** A PLCL membrane fixation with screw pins. **F** Wound closure

Study participants who met inclusion criteria were determined after preoperative examination and received GBR operation (Fig. 3). After administering local anesthesia, a crestal incision was made along the alveolar ridge, followed by mucoperiosteal flap elevation to expose the target site. Decortication was performed to induce bleeding, and carbonate apatite granules (Cytrans Granules®; GC Corporation, Tokyo, Japan) were grafted at the target site using saline, which was employed solely to facilitate handling and to avoid the influence of additional biological agents, thereby allowing evaluation of the material’s intrinsic regenerative potential. The grafted area was covered by a PLCL membrane (Cytrans Elashield®; GC Corporation, Tokyo, Japan), which was trimmed to fit GBR site. This membrane was fixed with small screw pins and/or sutures. Releasing incisions were performed when necessary to achieve adequate flap mobilization and tension-free closure. Wound closure was completed with sutures, and postoperative antibiotic and anti‑inflammatory analgesics were prescribed for three days, and additional medication was prescribed thereafter when clinically necessary.

### Postoperative procedures

Study participants were asked to visit the hospital at 10 ± 4 days, 1 month ± 7 days, and 3 months ± 7 days postoperatively for evaluation of postoperative conditions (Fig. 2). If any complications were identified during the visit, they were documented and managed according to their clinical status. A CT scan using the same radiopaque marker was performed at 6 ± 1 months after the GBR procedure to assess bone morphology.

### Radiographic evaluation

**Fig. 4:**
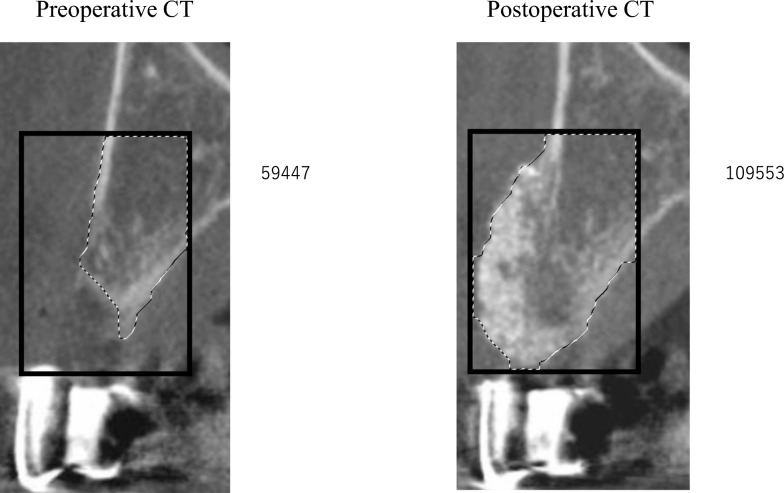
Comparative analyses of preoperative and postoperative CT images of the grafted site. Pre- and post-operative sites were aligned as closely as possible using radiopaque markers, and crestal bone width and grafted area outlined with dotted lines were measured using image analysis software

To evaluate the effectiveness of GBR, radiographic analysis was performed using preoperative and postoperative CT scans. Placement of the same radiopaque marker at the GBR site aided in aligning the scans for comparison. The alignment of the pre‑ and postoperative CT datasets was performed manually by one experienced examiner, and the results were independently verified by a second examiner. Using dedicated image analysis software, cross-sectional slices perpendicular to the alveolar ridge were selected at the center of the grafted region. Initially, crestal bone widths before and after surgery were statistically compared using dedicated image analysis software (AquariusNet Viewer, version 4.4.13.P5; TeraRecon Inc., Durham, NC, USA). Subsequently, CT images were exported and imported into Adobe Photoshop (version 26.11; Adobe Inc., San Jose, CA, USA), image analysis software. To facilitate standardized comparison, the preoperative cross-sectional bone area was defined as 100%, and the postoperative area was expressed as a percentage relative to this baseline (Fig. 3). This method enabled standardized evaluation of bone augmentation by referencing the preoperative area as 100%, thereby reducing anatomical variability among patients. Statistical analysis was performed to evaluate changes in cross-sectional bone area before and after guided bone regeneration. The distribution of data was assessed using the Shapiro–Wilk test. For normally distributed variables, results were expressed as mean ± standard deviation (SD), and comparisons between preoperative and postoperative measurements were performed using the paired t-test. For non-normally distributed variables, results were expressed as median and interquartile range (IQR), and comparisons were performed using the Wilcoxon signed-rank test. A p-value of < 0.05 was considered statistically significant. Statistical analyses were conducted using JMP Student Edition 18.2.1 (JMP Statistical Discovery LLC, Cary, NC) (Fig. 4).

## Results

### Study participants and grafted sites

This study was initially approved to begin in December 2019 and was scheduled for completion in September 2021. However, due to interruptions caused by the COVID-19 pandemic, the study period was extended, and it was officially concluded in December 2024. During this study period, a total of 29 patients were recruited as study participants after obtaining written informed consent. Following CT examination, ten participants were excluded due to sufficient bone volume, which did not meet the inclusion criteria for GBR, and two were excluded due to systemic conditions. Additionally, one participant withdrew from the study due to a protocol deviation identified during the early phase of data collection. Ultimately, 18 sites from 16 participants were included in the final statistical analysis.

Table 2 summarizes the characteristics of the study participants, the grafted sites and the cause of extraction. included in the analysis. The mean age and standard deviation were calculated following the Shapiro–Wilk test for normality. Among the 16 participants, five were male and 11 were female. Of the grafted sites, five sites in four participants were located in the maxilla and 13 sites in 12 participants in the mandible. Furthermore, two sites were in the incisor region, 10 in the premolar region, and six in the molar region.

**Table 2:** Patient characteristics and grafted site information

Study participant	Site	Cause of extraction	Summary
P 01: 68 years, female	21	Root fracture	**Mean age (SD)** 64.8 (8.5) **Sex** Male: 5, Female: 11 **Jaw** Maxilla: 4 (5 sites) Mandible: 12 (13 sites) **Tooth position** Incisor: 2 sites Premolar: 10 sites Molar: 6 sites
P 02: 75 years, female	44	Peri-implantitis	
P 03: 62 years, male	36	Root fracture	
	37	Periodontitis	
P 04: 68 years, male	24	Apical periodontitis	
	25	Apical periodontitis	
P 05: 70 years, female	15	Root fracture	
P 06: 55 years, female	46	Periodontitis	
P 07: 49 years, female	35	Root fracture	
P 08: 71 years, male	35	Root fracture	
P 09: 53 years, female	42	Root fracture	
P 10: 74 years, female	36	Root fracture	
P 11: 53 years, female	45	Root fracture	
P 12: 70 years, female	36	Unknown	
P 13: 61 years, male	34	Peri-implantitis	
P 14: 73 years, female	14	Root fracture	
P 15: 72 years, female	45	Apical periodontitis	
P 16: 62 years, male	36	Root fracture	

SD: standard deviation

### Surgical and postoperative outcomes

All GBR procedures were performed in accordance with the study schedule, using carbonate apatite granules mixed with saline and covered with PLCL membranes by four experienced clinicians, including board‑certified implant specialists. Although some teeth exhibited chronic inflammatory changes, no marked bone sclerosis was observed on preoperative CT, and all extraction sockets demonstrated uneventful healing prior to GBR. Carbonate apatite granules are available in two particle sizes (S and M size). In this study, the S size was used in most cases, while the M size was applied in five sites of five patients (P03, 08, 12, 14 and 16). An exploratory comparison showed no noticeable differences in graft volume between cases with or without fixation screws or releasing incisions. These procedures were performed based on intraoperative judgment, and no systematic variation in grafting amount was identified. No major complications were observed during the GBR procedures. Releasing incisions were performed in 13 out of 16 participants to achieve tension-free closure.

Regarding the postoperative course, dehiscence was observed in 6 of 18 cases (33.3%) at 2 weeks postoperatively, and in 4 of 18 cases (22.2%) at 1 month. No cases of infection were observed at each time point. At 3 and 6 months postoperatively, neither dehiscence nor infection was observed in any of the 18 evaluated sites.

### Radiographic analyses

All GBR sites were compared between pre- and post-operative CT images. Table 3 presents the outcomes of CT analyses. Following inclusion criteria, the width of crestal bone were evaluated pre- and post-operatively. Statistical analysis revealed that GBR using carbonate apatite granules and covered with PLCL membranes increased crestal bone width significantly (*P* < 0.01, paired t-test). In addition, CT-based evaluation of cross-sectional bone area demonstrated a significant increase postoperatively compared to baseline (*P* < 0.01, paired t-test). These results suggest that the combination of carbonate apatite granules and PLCL membranes effectively promoted bone volume augmentation.

**Table 3:** Pre‑ and postoperative CT measurement results for each patient and overall summary

Study participant (site)	Crestal bone width (cm)		Postoperative bone area (% of preoperative)
	Preoperative	Postoperative	
P 01 (21)	3.4	6.3	105.6
P 02 (44)	4.9	12.3	129.8
P 03 (36)	3.6	8.9	101.0
(37)	3.6	13.4	144.0
P 04 (24)	3.8	4.5	95.0
(25)	3.8	4.3	112.0
P 05 (15)	4.6	8.7	104.3
P 06 (46)	3.0	8.4	120.2
P 07 (35)	1.7	6.5	135.9
P 08 (35)	3.8	12.7	189.3
P 09 (42)	2.5	4.8	105.5
P 10 (36)	1.3	8.0	139.2
P 11 (45)	2.2	8.1	131.3
P 12 (36)	2.6	5.5	103.4
P 13 (34)	4.5	10.3	133.4
P 14 (14)	3.8	8.8	144.6
P 15 (45)	3.7	6.8	107.1
P 16 (36)	3.3	9.8	156.6
Mean (SD)	3.4 (1.0)	8.2 (2.8)	125.5 (24.2)
P-value	< 0.01*		< 0.01**

SD: standard deviation. *paired t-test, **Comparison with preoperative bone area (100%), paired t-test

## Discussion

This prospective clinical study evaluated the effectiveness of GBR using a combination of carbonate apatite granules and PLCL membranes. Radiographic analysis demonstrated a statistically significant increase in crestal bone width and cross-sectional area following GBR. As a minor complication, wound dehiscence was observed in some cases during the early postoperative period. However, no major complications or infections were noted throughout the healing phase. The lack of major complications may be attributed to the fact that all procedures were performed by experienced surgeons, despite the absence of a formal standardization protocol. These findings suggest that the use of carbonate apatite granules and PLCL membranes may offer a predictable and biocompatible approach for bone augmentation, although we were unable to evaluate potential differences in outcomes between the two criteria (two morphologies), because the number of cases in each morphological category was limited and unevenly distributed. However, Cases with smaller postoperative bone augmentation tended to involve flat alveolar bone surfaces, where horizontal augmentation was technically more demanding. Conversely, larger augmentation ratios were generally observed in defects with concave or cavity‑like morphologies that provided better containment for the graft material. These tendencies indicate that defect morphology could play a role in the variability of augmentation outcomes. Further investigations with adequately powered, morphology‑based comparisons will be necessary to confirm these findings.

Carbonate apatite granules are synthetic bone graft materials designed to closely mimic the mineral composition of natural bone, particularly in terms of carbonate content, and their osteoconductive nature facilitates cellular attachment, proliferation, and bone matrix deposition, while their gradual resorption profile allows for sustained space maintenance during the healing phase [22, 23]. In Japan, carbonate apatite has been clinically approved and widely utilized in implant dentistry, offering a predictable alternative to autogenous or xenogeneic grafts [17–19]. Although histological evaluation was not performed in the present study, previous literature provides insight into the tissue response to CO₃Ap granules approximately 6 months after augmentation [18, 19]. Histological analyses have shown that newly formed bone accounts for around 40% of the augmented area, while residual CO₃Ap granules remain at approximately 13–15%. Importantly, the granules are typically surrounded by new bone without intervening fibrous tissue, and only minimal inflammatory cells or foreign‑body giant cells are observed. CO₃Ap granules are gradually resorbed and replaced by new bone, with osteoblasts frequently observed on their surfaces. These findings indicate that CO₃Ap maintains structural stability during the healing period while supporting progressive bone maturation, which is consistent with the clinical observation that implant placement is feasible despite the presence of residual granules.

The PLCL membrane used in this study is a synthetic copolymer composed of poly(lactic acid) and poly(caprolactone), forming a bilayer structure with high biocompatibility and controlled degradation [20, 21]. Its hydrophilic surface enhances handling and adaptation to the grafted site, while its mechanical integrity supports space maintenance without the rigidity associated with non-resorbable membranes. Importantly, the resorbable nature of PLCL eliminates the need for a second surgical intervention, reducing patient burden and improving procedural efficiency [4].

In this study, the combination of carbonate apatite granules and PLCL membranes offers a synergistic balance of biological compatibility, mechanical stability, and surgical manageability, which may have contributed to the observed bone augmentation and low complication rates. This would be attributed to the osteoconductive properties of carbonate apatite and the functional characteristics of PLCL membranes, including sufficient space maintenance when used in combination with carbonate apatite, barrier properties that prevent soft tissue ingrowth, and resorbability that allows for subsequent bone formation. These findings are consistent with previous reports demonstrating the clinical efficacy and biocompatibility of carbonate apatite granules and PLCL membranes in GBR procedures [24–26].

Notably, although membrane exposure was observed in several cases during the early postoperative period, no infections were reported at any time point. This suggests that these materials were well tolerated by the surrounding tissues and that the PLCL membrane maintained its barrier function even under compromised soft tissue conditions. Its resorbable nature may have further contributed to tissue compatibility and the absence of infection, even in cases of early exposure. These findings highlight the clinical potential of the combination of carbonate apatite and PLCL membranes in achieving predictable GBR outcomes with minimal biological risk and procedural burden.

Previous reviews on guided bone regeneration have emphasized that successful outcomes depend largely on the membrane’s ability to provide stable space maintenance, ensure adequate barrier function, and minimize the risk of bacterial contamination [1–5, 16]. Materials that maintain structural integrity during healing while resisting infection are therefore considered advantageous in GBR procedures. Both CO₃Ap granules and the PLCL membrane used in this study are fully synthetic and domestically approved materials. CO₃Ap has demonstrated favorable osteoconductive capacity [17–19, 22, 23], while the resorbable dual‑layer PLCL membrane provides stable space maintenance without the biological risks associated with animal‑derived products [20, 21, 24, 25]. These characteristics suggest potential advantages in terms of safety and infection control. Although direct comparisons with other graft–membrane combinations are limited by differences in clinical conditions, the favorable outcomes observed in this study indicate that this synthetic approach may offer a clinically effective option for GBR. Furthermore, although direct comparison with previous studies is challenging due to differences in defect morphology, graft materials, and imaging protocols, the amount of horizontal bone gain observed in this study appears to be within the range commonly reported for GBR procedures using particulate grafts and resorbable membranes. Previous clinical studies have typically demonstrated horizontal augmentation of approximately 2–4 mm [1–9], and the present findings fall within this expected range, suggesting that the combination of CO₃Ap granules and a PLCL membrane provides clinically comparable regenerative performance. However, future studies with larger sample sizes will be needed to further validate its benefits.

Despite the promising results, several limitations should be acknowledged. The study employed a single-arm design without a control group, which limits direct comparison with other GBR materials or techniques. Additionally, the sample size was relatively small, and the follow-up period was limited to six months. Future studies with larger cohorts and longer observation periods are recommended to validate the long-term stability and clinical outcomes of GBR using synthetic materials. Moreover, the evaluation was based primarily on radiographic measurements derived from two-dimensional cross-sectional CT images, which may not fully capture the three-dimensional nature of bone regeneration. In addition, the manual alignment procedure was not evaluated using a formal reproducibility test such as an intraclass correlation coefficient. Ideally, more precise selection and alignment of pre‑ and postoperative three‑dimensional assessment that includes mesiodistal morphology might have provided a more comprehensive evaluation of defect volume. However, to simplify participant selection and ensure consistency, we adopted a two‑dimensional approach based on buccal or lingual morphology. As discussed, volumetric assessment would have strengthened the analysis, but the present study focused on bone width and cross‑sectional area. Finally, immediate postoperative CT evaluation was not performed in this study. Although such imaging could have provided detailed information on the initial graft contour, membrane position, and space maintenance, routine CT acquisition immediately after GBR is not commonly implemented in clinical practice due to concerns regarding additional radiation exposure and limited impact on short‑term clinical decision‑making. The absence of immediate CT data prevented us from assessing the early stability of the graft and membrane configuration, which may influence subsequent healing dynamics. Future studies incorporating immediate postoperative imaging would allow a more comprehensive understanding of the relationship between initial graft morphology and long‑term augmentation outcomes. Future investigations incorporating volumetric analysis, immediate postoperative CT observation as part of early imaging, histological evaluation, or outcomes of subsequent implant placement would provide a more comprehensive understanding of the regenerative potential and clinical relevance of this approach.

## Conclusions

GBR using a combination of carbonate apatite granules and PLCL membranes demonstrated favorable clinical outcomes, including significant bone augmentation and low complication rates. The combination of these synthetic materials may provide a predictable and minimally invasive approach to GBR in implant dentistry.

However, the findings should be interpreted in light of several limitations, including the single-arm study design, limited sample size, short-term follow-up, and the use of two-dimensional radiographic evaluation. Future studies incorporating volumetric imaging, histological evaluation, and long-term implant outcomes are favorable to further validate the clinical applicability and regenerative potential of this approach.

## Data Availability

The datasets used and/or analyzed during the current study are available from the corresponding author on reasonable request.

## Abbreviations

CRB: Clinical research review board
CT: Computed tomography
ePTFE: Expanded polytetrafluoroethylene
GBR: Guided bone regeneration
jRCTs: Japan Registry of Clinical Trials
PLCL: Poly(lactic acid/caprolactone)
SD: Standard deviation
